# Drug pricing and reimbursement decision making systems in Mongolia

**DOI:** 10.1186/s40545-017-0098-6

**Published:** 2017-02-27

**Authors:** Gereltuya Dorj, Bruce Sunderland, Tsetsegmaa Sanjjav, Gantuya Dorj, Byambatsogt Gendenragchaa

**Affiliations:** 1 Department of Clinical Pharmacy and Pharmacy Administration, School of Pharmacy and Biomedicine, Mongolian National University of Medical Sciences, Ulan Bator, Mongolia; 2 School of Pharmacy, Faculty of Health Sciences, Curtin University of Technology, WA Perth, Australia; 3 School of Pharmacy and Biomedicine, Mongolian National University of Medical Sciences, Ulan Bator, Mongolia; 4 Department of Epidemiology and Biostatistics, School of Public Health, Mongolian National University of Medical Sciences, Ulan Bator, Mongolia; 5 ISPOR Chapter Mongolia, Bayanzurkh district, 76-26, 13374 Ulaanbaatar, Mongolia

**Keywords:** Drug pricing policy, Reimbursement, Cost, Pharmaceuticals, Mongolia

## Abstract

**Background:**

It is essential to allocate available resources equitably in order to ensure accessibility and affordability of essential medicines, especially in less fortunate nations with limited health funding. Currently, transparent and evidence based research is required to evaluate decision making regarding drug registration, drug pricing and reimbursement processes in Mongolia.

**Objective:**

To assess the drug reimbursement system and discuss challenges faced by policy-makers and stakeholders.

**Methods:**

The study has examined Mongolian administrative documents and directives for stakeholders and analysed published statistics. Experts and decision-makers were interviewed about the drug pricing and reimbursement processes in Mongolia.

**Results:**

Decisions regarding Mongolian drug registration were based on commonly used criteria of quality, safety, efficacy plus some economic considerations. A total of 11.32 billion Mongolian National Tugrugs (MNT) [5.6 million United States Dollars (USD)] or 12.1% of total health expenditure was spent on patient reimbursement of essential drugs. The highest reimbursed drugs with respect to cost in 2014 were the cardiovascular drug group. Health insurance is compulsory for all citizens; in addition all insured patients have access to reimbursed drugs. However, the decision making process, in particular the level of reimbursement was limited by various barriers, including lack of evidence based data regarding efficacy and comparative cost-effectiveness analysis of drugs and decisions regarding reimbursement.

**Conclusions:**

Drug registration, pricing and reimbursement process in Mongolia show an increasing trend of drug registration and reimbursement rates, along with lack of transparency. Limited available data indicate that more evidence-based research studies are required in Mongolia to evaluate and improve the effectiveness of drug pricing and reimbursement policies.

## Introduction

Mongolia is an East-Asian country bordered with Russia and China. It is the 19th largest country in the world with an estimated area of 1,566,460 km^2^. After the collapse of the Soviet Union, Mongolia has undergone radical change in financial support for health, education and social security. Despite steady economic growth with promising developments occurring in the last 15 years, recent statistics show that infectious diseases are no longer the leading cause of morbidity, instead lifestyle, behaviour –dependent diseases, including circulatory system diseases, cancer and injuries have become the leading causes of mortality and morbidity [1].

During the years of socioeconomic transition in Mongolia, total health expenditure (THE) as a share of Gross Domestic Product (GDP) has increased from 3.3% (1995) to 5.4% by 2010 [2, 3].

Despite existing government regulations, inappropriate use of medicines [4] and high drug costs are evident in Mongolia [5]. Although illegal, people can purchase prescription medicines, including antibiotics without prescription from some private pharmacies [6, 7]. Previous reports have indicated lower out of pocket (OOP) expenses with about 10% of outpatients and 16% of inpatients paying OOP fees for hospital visits in 2011 [8]. However, the study did not specify whether these fees were for medicines or treatment.

### Overview of the Mongolian health sector

The Mongolian health sector is regulated by the Ministry of Health (MOH), the Ministry of Finance (MOF), the Ministry of Human Development and Social Welfare (MOHDSW), the Ministry of Education and Science (MOES), the regulating agency -General Agency for Specialised Inspection (GASI) and former government implementing agencies such as the Department of Health (DOH), the Department of Physical Culture and Sport (DOPS), and the city/aimag (provincial) health departments.

The MOH and MOHDSW are the third-party payers involved in purchasing and resource allocation in health care. The government health budget is managed by the MOH, and the Health Insurance Fund is managed by Social Insurance General Office (SIGO- under the MOHDSW). The fund for social health insurance (HI) is a single national insurance fund that uses its local branches to collect revenue and pay for insured care and it has been a stable source of health financing in Mongolia since 1990s. In addition to having government agency status, the SIGO is also overseen by the Social Insurance National Council (SINC) appointed by the State Great Khural.

Despite having a low share of THE, it is the only health financing mechanism that exercises some elements of contracting and purchasing. The government is the dominant player in making HI decisions therefore it is used as a substitute for the government budget.

### Pharmaceutical sector in Mongolia

**Fig. 1:**
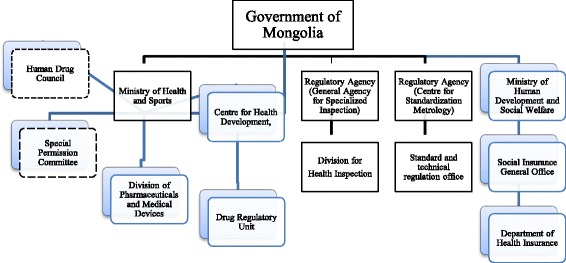
Organizational structure of the Mongolian pharmaceutical policy and regulation. Note: Human Drug Council and Special Permission Committees are expert professionals

**Fig. 2:**
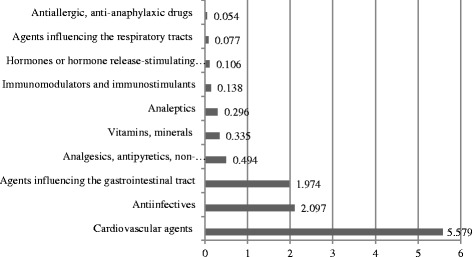
Proportion of reimbursed drugs, by pharmacological classification (Billion MNT), 2014 (adapted from Health Indicators of Mongolia, 2014)

The Division for Pharmaceutical and Medical Devices of the MOH is responsible for oversight of the main functions of the pharmaceutical sector policy, regulation and coordination. The Division for Health Inspection of the General Agency for Specialized Inspection ensures compliance with major laws and legislation as it relates to quality assurance and distribution inspection. A Human Drug Council consisting of experts in the field and representatives of all relevant ministries leads the pharmaceutical sector, particularly in the development of standards, guidelines and procedures, including drug registration. The Drug Regulatory Unit is responsible for technical work for the Human Drug Council including the registration of medicines and medical devises, licensing of specialists and providers, issuance of import and export licenses, monitoring and reporting adverse drug reactions, monitoring medicines marketing and advertisement, promoting rational use of medicines and developing a national pharmacopeia and standards. In addition, a Special Permission Committee of the MOH monitors the functional activities of drug producers and grants approval for manufacturing, importing and selling drugs in Mongolia (Figs. 1 and 2).

The drug reimbursement decision is approved by the Health Insurance Fund (HIF), which is incorporated within the General Department of Social Insurance and it is regulated by the Ministry of Human Development Social Welfare of Mongolia.

The Drugs Act of Mongolia was promulgated in 1998 and aimed to ensure good quality, effective and safe drugs were available to the Mongolian population. Following the Drug Act, the National Drug Policy of Mongolia (NMPM) was adopted in 2000, revised and approved by the State Great Khural in 2014. Ministerial legislation corresponding to reimbursement includes the Health Insurance Law, approved by the State Great Khural 2015 and the Resolution on the List of Essential Medicines to be reimbursed by the HIF, approved by the National Committee on Social Insurance of Mongolia in 2016.

The Policy regulates the procurement, manufacturing, financing, quality assurance, distribution, and appropriate use of drugs. In order to ensure the availability of the most essential medicines at all levels, the government has adopted an Essential Drugs List, developed on the basis of recommendations by the World Health Organization (WHO). This List has been available since 1991 and it has been revised several times with the seventh being completed in 2014 [9].

The procurement of health products including medicines for only public health facilities (hospital pharmacies) is regulated by the Law on Public Procurement and Guidelines approved by the MOH. Tertiary-level health facilities and aimag health departments have their own tendering committees. Ulaanbaatar city carries out a tender for all its district hospitals in the urban area. Medicines procured and sold at private retail pharmacies are supplied by wholesaling companies, however the government has no regulation on price.

The Mongolian pharmaceutical sector is predominantly dependent on its manufacturing and private business organizations due to 100% privatization of all pharmaceutical wholesalers. Mongolia was defined as a low-income country with THE of 556 billion MNT or 2.6% of GDP in 2013 [10], hence evidence based decision making and optimum use of available resources is of high importance.

### Drug distribution system in Mongolia

Most of the imported drugs sold in Mongolia come from the Russian Federation, China, India and other eastern European countries. Drug supply companies and pharmacies must be licensed in order to undertake drug supply functions. With the accelerating growth of the private sector, the drug supply business has proven to be very successful. Currently, 1190 pharmacies are operating all over Mongolia and 75% of the private pharmacies have one or two branches. There were 306 pharmacies working under the drug revolving fund (DRF) initiative [1]. According to the latest report, 24 drug factories were officially permitted to operate as drug manufacturers and their share was approximately 12% (66 Billion MNT) of the total local pharmaceutical market [11]. There were 591 new drugs (salts or dosage forms), 48 raw materials registered and the registration period was extended for a total of 428 drugs in 2014 [11].

The latest survey on medicines prices and affordability in Mongolia was completed using WHO methodology in 2010 and it reported that the affordability of the lowest priced generics and most sold generics in the public sector was good for most conditions, with standard treatment costing 1 days' wage or less. Treatments costing more than 1 days' wage of the lowest paid unskilled government worker included a pack of 30 amlodipine tablets to treat hypertension (1.6 days) [5]. In the private sector, the majority of treatments cost less than the daily wage of the lowest paid government worker when the lowest price generics were used. Hypertension treated with enalapril (10 mg cap/tab) for 30 days costs 2.5 days’ wages, simvastatin (20 mg cap/tab) 30 days 2.7 days’ wages are clearly unaffordable even when generics are used.

The price of medicines has not been regulated by the Government since 1997 and a 10% value-added- tax (VAT) was introduced in 1999. Taxes, duties and other government charges applied to medicines include 5% customs duty for imported medicines. In the private sector, add-on costs represent 90.1% of the final patient price for imported originator brands, 115.5% for imported generics, and 74.4% for locally produced generics. Registration fees do not differ between originator brands and generic equivalents. However, registration fees are lower for locally produced than for imported medicines; hence the government encourages local production.

There are no public pharmacies, except those located in public hospitals whose service is limited to inpatients. Furthermore, the government controls dispensing fees for reimbursed essential medicines and some data are available to support the insurers view on the dispensing fee.

The drug reimbursement system and health insurance scheme have been developed since 1994. The latest revision of the list for reimbursed essential drugs was approved by the SHI in 2016 [12]. The main purpose was to expand the range of health insurance benefits and ensure greater access to essential drugs in primary health-care services by the insured population which is compulsory for all population groups [12].

To date, insured ambulatory patients have access to a total of 134 essential drugs partly reimbursed by the Health Insurance (HI) providing that these drugs were prescribed by a family doctor (in urban settings), village (soum) hospital doctor or district (bagh) feldsher (in rural settings) and dispensed by a HI designated or contracted pharmacy.

A list containing the cost of certain essential drugs is also available once it has been approved by the HI. This list gives the maximum price level for certain drugs provided to insured ambulatory and stipulates how much of this cost should be covered under HI. A decision to include the drug in the reimbursement list is based on mortality and morbidity rates for the last 2 years and drug consumption listed by pharmacological classification. No further information of specific analysis for reimbursement decision making was available.

Drug reimbursement funding is an important expenditure to ensure the budget is used optimally. However, there is a lack of detailed information in regards to drug pricing and reimbursement decisions in Mongolia. Considering the importance of clear and transparent processes for drug pricing and government funded reimbursement, this study has aimed to examine the pharmaceutical drug pricing and reimbursement decision making in Mongolia.

## Methods

### Documents relevant to drug reimbursement

Existing documents regarding drug reimbursement, selection and procurement of essential medicines, appropriate use of medicines including regulations enacted by the Mongolian Government, legislative documents and published internal regulations of the Human Drug Council, Ministry of Health of Mongolia (MOH), Pharmaceuticals and Medical Devices Division, (MOH), Social Health Insurance Office (SHIO), and the Division of Finance and Insurance, the Ministry of Finance, Mongolia (MOF), including approved State Policy on Medicines and Medical Devices (2014), Health Law (2010), regulation to reimburse essential medicines by the Health Insurance, approved by the Health Insurance Subcommittee, National Social Insurance Committee, #03, 2011, anupdated list of reimbursable essential medicines, Health Insurance Subcommittee, National Social Insurance Committee (2013.07.31), General requirement for pharmacies in Mongolia, MNS 260:2011,#13 Regulation for registration of medicines, raw materials, biologically active compounds in Mongolia, approved by the Health Minister, 2015 were reviewed by all authors in this study. More documents in relation to the statistics and published data related to drug pricing and drug reimbursement, published and unpublished annual reports reimbursement of medicines, including Mongolian statistical annual report, National Statistical Office, Annual report of Health Insurance Office, Survey of medicine prices, availability, affordability and price components in Mongolia, 2012 [5] were collected and reviewed.

In addition, websites of several organisations and agencies including drug regulatory authorities were examined for relevant information and reports. The search was complemented by hand searches of bibliographies and, in the case of doubt, by telephone and email communication with the institutions themselves.

### Interviews

In order to collect more detailed information on the decision making process and evaluation methods, key stakeholders including the authorities and officers in charge of drug registration, procurement and reimbursement including the Head of Pharmaceuticals and Medical Devices Division, Health Insurance Office, Head of Allocation and Monitoring of Health Budget, Ministry of Finance, Human Drug Council Member, Officer in charge for drug procurement services, policy implementation, MOH were interviewed. The interview consisted of questions regarding information about the respondent [13] and reimbursement, including the (i) assessment, (ii) appraisal and (iii) decision making processes. Assessment included the quantification of the clinical, pharmacotherapeutic efficacy and pharmacoeconomic value of a drug. Appraisal sought to gauge society’s willingness to pay for a drug by weighing assessment outcomes against other (societal) criteria which reflect health system objectives. Decision making was defined as a value judgement from a broader societal perspective, considering the health system policy objectives as well as non-health care related objectives.

The interview guide (in Mongolian) was piloted with four potential respondents to ensure the validity; technical functioning, relevance and understanding of the questions. No major omissions were identified. These responses were not used further in the study.

As Mongolian versions of the documents are available, the study engaged two professional translators to complete translations from Mongolian to English and vice-versa to assure accuracy and minimize any possible bias. These translators were unknown to each other [14]. The author made adjustments resulting from any inconsistencies. (Data are available upon request from the corresponding author).

### Data analysis

Standard descriptive statistics were used to summarize demographic data and responses to the interviews. Questions regarding the frequency of assessment, appraisal and decision making were answered. All documents were analysed in a qualitative matter and according to an analytic informational framework that had been developed in advance. Results were summarised narratively and presented in a related manner. Comparisons between groups were performed using the Chi-square test, logistic regression or Kruskal–Wallis test with a pairwise comparison as appropriate. A *p* value of <0.05 was taken as being significantly different.

## Results

### Drug registration process in Mongolia

All drugs used in the country are registered once agreement and authorization by the Human Drug Council has been given. The General Agency for Specialized Inspection (GASI) is in charge of ensuring that only registered drugs enter the market for public use. Locally manufactured drugs are registered for 2 years, whereas imported drugs are given a 4-year (regular registration) or 5-year (fast-track registration) period. The drugs should be of a good quality, compliant with good manufacturing practice (GMP) regulations to qualify for registration. The applicant must prepare documents including data regarding efficacy, safety and adverse events, comparative efficacy with similar drugs, approval history, contraindications, warnings, precautions, monitoring parameters, pharmacokinetics, patient compliance and information of cost, insurance and freight (CIF) cost for registration.

All medicines should be registered in the State Medicines Register which is divided into two categories as medicines available with and without prescription (over-the-counter, OTC).

### Drug reimbursement process in Mongolia

Detailed analysis of HI statistics indicates that 11.32 Billion MNT (5.59 Million USD) was spent on reimbursement of essential drugs, and that half of the reimbursement was spent on cardiovascular agents (5.579 Billion MNT) in 2014 (Table 1). Considering the health indicators, the prevalence of cardiovascular diseases is listed as priority causes of mortality and morbidity [1].

**Table 1:** Drug categories and reimbursement data for essential drugs for the year 2014

Number	Drug	Dosage	Dosage form	Package size	Maximum retail price MNT	Reimbursement value (%/)	Reimbursement from HIF/MNT/	Total quantity (thousand)	Total reimbursed/mlnMNT/
	1. Analgesics, antipyretics, non steroidalantiinflammatory drugs								
1	Acetyl salycilic acid	100 mg	tablet	100	1500	66,7	1000	1,145,655	11.4
				10	3000	50	1500	4,041,900	4.9
				30	2000	50	1000	432,918	1.4
		150 mg		10	3500	57,1	2000	364,000	0.6
		300 mg		10	5000	60	3000	14,472	4.3
2	Diclofenacsodium	25 mg	tablet	10	750	66,7	500	22,714	4.6
		50 mg		10	750	66,7	500	195,847	36.7
		75 mg		10	7500	53,3	4000	0	0
		100 mg		10	1500	66,7	1000	0	0
		50 mg	suppositorium	10	1500	66,7	1000	9912	0.9
		100 mg		2	2500	80	2000	45,015	45.9
		20 гp	gel	1	4000	50	2000	0	0
3	Ibuprofen	200 mg	tablet	12	5000	60	3000	115,355	28.8
		400 mg		10	1500	66,7	1000	996,263	99.3
		100 ml	syrup	1	8500	58,8	5000	41,235	205.6
4	Indomethacin	25 mg	tablet	30	1800	55,6	1000	51,436	2.6
				20	1200	83,3	1000	0	0
5	Paracetamol	500 mg	tablet	10	250	40	100	64,506	0.6
				12	1200	50	500	3373	0.1
		100 mg	suppositorium	10	250	57,1	2000	13,264	2.6
		100 ml	suspension	1	1000	61,5	4000	1015	4.1
		3%-90 ml	syrup	1	3500	50	3000	529	1.6
6	Tramadol	50 mg	tablet	10	3300	60,6	2000	88,245	17.6
	Sub total							7,647,654	473.8
	5.2 Antiinfective agents								
	*5.2.1 Beta-lactame antibiotics*								
25	Amoxicillin	125 mg	syrup	1	4000	50	2000	3547	7.4
		250 mg		1	7000	57,1	4000	293,886	30.1
		125 mg		10	5000	60	3000		
		250 mg		10	1500	62,5	1000	293,886	30.1
		500 mg		10	2000	50	1000	5,062,806	56.9
26	Amoxicillin + clavulanic acid	100 ml	syrup	1	10,000	50	5000	1799	8.9
		156.25 mg/5 ml		1	8100	61,7	5000	5179	25.8
		228.5 mg/5 ml		1	7200	69,4	5000	602	3
		125 mg/60 ml		1	5000	80	4000	1980	7.9
		156.25 mg	tablet	10	7500	60	4500	5179	25.8
		312.5 mg		10	10,000	50	5000		
		375 mg		16	15,200	52,6	8000		
		625 mg		10	16,000	62,5	10,000	442,351	439.8
27	Phenoxymethylpenicllin	250 mg	tablet	10	850	58,8	500	6800	0.3
28	Cefadroxil	500 mg	capsule/tablet	10	3800	65,8	2500	8244	2.1
	Sub-total							6,126,259	638.4
	5.2.2 Other antiinfectiveagents								
29	Clarithromycin	500 mg	tablet	14	21,000	47,6	10,000	494,548	351.9
		250 mg		14	15,400	51,9	8000	145,636	83.2
30	Doxycycline	100 mg	capsule/tablet	10	2150	69,7	1500	21,097	3.2
31	Metronidazole	250 mg	tablet	10	350	85,7	250	147,898	3.7
		500 mg		10	1500	71,4	1000	67,655	10.8
		500 mg	suppositorium	6	3000	66,7	2000	2199	0.7
32	Chloramphenicol	250/500 mg	capsule	10	1200	83,3	1000	145,020	14.3
33	Sulfamethoxazol + trimethoprim (trimexasol)	480 mg	tablet	10	1000	50	500	85,087	43.2
		240 mg/5 ml-60 ml	suspension	1	2500	80	2000	949	3.1
34	Ciprofloxacin	250 mg	tablet	10	3000	66,7	2000	98,752	46
		500 mg		10	4500	66,7	3000		
35	Azithromycin	100 mg-5 ml	suspension	1	11,600	69	8000	7245	58
		250 mg	capsule	10	9000	55,6	5000	1,134,303	63
		200 mg/5 ml-20 ml	suspension	1	15,000	66,7	10,000		
	Sub total							2,350,389	681
	*5.3 Antifungal agents*								
36	Griseovulvin	125 mg	capsule	10	800	62,5	500	868	0.04
37	Fluconazol	50 mg	capsule	7	9100	54,9	5000	155,658	111.1
38	Clotrimasol	100 mg	vaginal tablet	6	3000	66,7	2000	38	0.08
		500 mg		1	3500	57,1	2000		
		20гp	cream	1	2500	80	2000	296	0.6
		15гp		1	2200	68,2	1500	84	0.1
		10гp		1	2800	71,4	2000	146	0.3
39	Nystatin	500,000 unit	Coated tablet	20	2500	80	2000	3620	3.6
		500,000 unit	Vaginal suppositorium	10	2800	71,4	2000	1704	0.3
		2,500,000 unit		10	2600	76,9	2000	10,475	2.1
	Sub total							172,889	118.3
	5.4 Antiviral agents								
40	Aciclovir	200 mg	tablet	20	4500	66,7	3000	120,741	19.5
		5%-5гp	ointment	1	3000	66,7	2000	2044	4.1
		5%-2гp	cream	1	12,500	80	10,000	1020	10.2
41	Ribavirin	200 mg	capsule	20	12,000	66,7	8000	5760	2.3
42	Lamivudin	150 mg	tablet	10	6000	66,7	4000	355	0.1
	Sub total							129,920	36.1
	6. Medication for migraine prophylaxis								
43	Propranolol	40 mg	tablet	50	3000	66,7	2000	40,677	1.6
	Sub total							40,677	1.6
	9. Drugs acting on blood								
	*9.1 Hematinic agents*								
49	Ferlatum	60 mg	tablet	10	1500	66,7	1000	7501	0.8
50	Ferrovitum	162 mg + 0.75 mg + 7.50 mg	capsule	30	5400	55,6	3000	121,772	12.2
51	Folic acid	5 mg	tablet	100	4000	50	2000	31,828	0.6
	Sub total							161,101	13.6
	10. Cardiovascular agents								
	*10.1 Drugs for* Angina pectoris, schemic heart diseases								
52	Atenololum	50 mg	tablet	30	3000	66,7	2000	330,066	21.9
		100 mg	tablet	10	2000	50	1000	31,812	3.2
53	Verapamilum	40 mg	tablet	50	3500	57,1	2000	16,038	0.6
54	Glycerilumtrinitratum	6.4мu	tablet	25	4750	63,2	3000	5082	0.6
55	Isosorbidumdinitratum	5 mg	tablet	20	900	55,6	500	80,812	2
56	Nifedipinum	10 mg	tablet	50	2500	80	2000	809,139	32.3
57	Inozinum	200 mg	tablet	50	2200	68,2	1500	1,250,837	37.4
	Sub total							253,786	98
	*10.2 Drugs for arrhythmia*								
58	Amiodarone	200 mg	tablet	30	7500	66,7	5000	50,865	8.5
	*10.3 Antihypertensives*								
59	Hydrochlorthiazidum	25 mg	tablet	30	3000	66,7	2000	7869	0.5
				10	1200	83,3	1000	6557	0.6
60	Methyldopa	250 mg	tablet	50	12,000	66,7	8000	60,237	9.6
61	Amlodipin	5 mg	tablet	30	16,200	61,7	10,000	1,578,754	525.8
		10 mg	tablet	30	30,000	50	15,000	6,135,264	3059.5
62	Enalapril	2.5 mg	tablet	20	3000	66,7	2000	25,525	2.5
		5 mg	tablet	30	3900	76,9	3000	310	0.1
		10 mg	tablet	30	5400	74,1	4000	1260	1.7
		20 mg	tablet	30	8100	61,7	5000	1580	2.4
63	Lozartan	25 mg	tablet	28	10,500	76,2	8000	190,397	54.4
		50 mg	tablet	28	14,000	71,4	10,000	1,105,003	393.7
		100 mg	tablet	28	28,600	52,4	15,000		0
64	Lizinopril	5 mg	tablet	14	5500	54,5	3000		0
		10 mg	tablet	14	7000	57,1	4000		0
		20 mg	tablet	14	8500	58,8	5000		0
	Subtotal							9,112,756	405.9
	*10.4 Heart failure*								
65	Digoxin	250mkg	tablet	30	3000	66,7	2000	6269	0.4
	Sub total							6269	0.4
	*10.5 Lipid lowering drugs*								
67	Simvastatin	10 mg	tablet	30	13,200	60,6	8000	65,260	17.4
		20 mg	tablet	30	22,000	45,5	10,000	322,778	107.6
		40 mg	tablet	30	36,000	50	18,000	587,264	352.3
	Sub total							975,302	477.3
	*14. Diuretics*								
77	Spirinolactone	25 mg	tablet	20	4200	71,4	3000	344,164	51.5
78	Furosemide	40 mg	tablet	30	800	62,5	500	23,333	38,410
	Sub total							346,497	38461.5
	21. Vitamins and minerals								
125	Ascorbucacidm	50 mg	tablet	10	200	50	100		0
126	Vitamin B complex	100 mg + 200 mg + 300mkg	dragee	60	3200	62,5	2000		0
		100 mg + 200 mg + 300mkg	coated tablet	10	1700	58,8	1000		0
		250 mg + 250 mg + 1000mkg	tablet	10	3600	76,9	2000	88,708	17.6
127	Calcium gluconate	500 mg	tablet	10	250	40	100	59,066	0.6
128	Calcium glycerphosphate	500 mg	tablet	10	1200	83,3	1000	18,354	0.2
129	Polivitamine	50 mg	dragee	50	300	33,3	100	78,739	0.2
		150 mg	syrup	1	8000	50	4000	66,666	264.4
		500 mg	Coatedtablet	10	1200	83,3	1000	18,794	1.9
130	Nicotinamide	50 mg	tablet	10	800	62,5	500	15,518	0.8
131	Pyridoxine	20 mg	tablet	10	400	75	300	740	0.02
132	Retinolumpalmitatum	20 mg	dragee	10	1100	45,5	500	18,678	0.9
		12,000 IU	capsule	10	900	55,6	500	10,868	0.5
133	Ergocalciferol	15,000 IU 1 ml	solution	1	4500	66,7	3000	553	1.7
134	Thiamine	50 mg	tablet		700	71,4	500	1150	0.1
	Sub total							88,708	17.6

A detailed analysis of reimbursed cardiovascular drugs indicated that the highest was for amlodipin (3,059,489,957 MNT) and the lowest was for enalapril (69,266 MNT). In terms of analgesics, antipyretics, non-steroidal anti-inflammatory drugs, the most reimbursed item was ibuprofen syrup 9,205,575,118 MNT) and the least dispensed item with reimbursement was paracetamol tablets (12 pack, 140,486 MNT) (Table 1).

The proportion of pharmaceutical expenditure has been relatively stable ranging from 12 to 14% of total health expenditure in the last 5 years, whereas the reimbursement provided by the HI has steadily increased over the last 6 years. The revised list of reimbursable essential medicines contained a total of 134 medications prescribed by a legal prescriber/physician the reimbursement level has ranged from 40 to 83.3% of the cost of the medicine. This list indicates the maximum retail price that a retail pharmacy can charge. This indicates that the government is fixing the total cost of these medicines charged by retail pharmacies.

The latest statistics indicate that the population coverage has increased to 99% which means essentially the whole population is covered for essential drugs [1].

Until 2014, there were no price control mechanisms specific to generic drugs, however the latest revision of the NDPM indicates that the maximum price of essential medicines shall be regulated by the Government [15]. Therefore the only price control is on reimbursed essential medicines in Mongolia.

## Discussion

This study has indicated that the Mongolian drug registration process is based on commonly used decision making criteria that are applied in many countries [13, 16]. These criteria include safety, efficacy and some economic aspects. Official reports and documents also have shown that Mongolian HI operates with some advantages, including a 92.2% of insurance coverage in Mongolia [2]. However, due to low payment in regards with salary (lowest: 192000 MNT or 101.3 USD) and pension (lowest: 145.200 MNT or 87 USD), the health care benefits of an estimated 1.33 million MNT or 798 USD per person annually are reported to be rather weak [17].

As acknowledged in many other countries, the drug reimbursement process is a very challenging task shared by various authorities with different interests [18]. The Ministry of Health of Mongolia works towards delivering quality health care and increased efficacy and accessibility of drugs to all patients, whereas the Ministry of Finance and Ministry of Human Development and Social Welfare aim to increase the health insurance coverage with limited health funding. However, HI involvement in the decision making process, the use of evidence-based data, including post marketing analysis, cost-effectiveness analysis are lacking. The different aspects and number of various institutions involved leads to complexity in the current system which can have a negative impact on health care delivery, including increased OOP payment for drugs. Obviously, this is a major point where the vast array of government departments and offices involved must make the system fragmented and avoid much duplication.

The funding source of pharmaceuticals in Mongolia was analysed and the non-government sources including the donor organizations and patient OOP, played a minimal role for total pharmaceutical expenditure (TPE) (4.8%) whereas government sourced fund (tax) was the highest (80.4%) [19]. No data are available on private health funds in Mongolia. Previous findings reported that OOP payments for health services have increased from 14.5% of the THE in 1995 to 41.4% in 2010. However, data in regards to OOP payments for pharmaceuticals were not available [19, 20]. The WHO reviewed the total expenditure on pharmaceuticals in different countries and compared by GDP. The median expenditure for pharmaceuticals in European region was 13% and Asian countries spent approximately 35% of total health expenditure on pharmaceuticals. However, the data represent both public and pharmaceutical expenditure [20]. The comparison of proportion of pharmaceutical expenditure in different regions indicated that Mongolia had the lowest public pharmaceutical expenditure (11.6%) [20].

It is widely accepted that modern health technology assessment methods should be used in such processes [21]. Successfully implemented health technology assessment programs including the evaluation of efficacy, comparative cost-effectiveness of medical interventions and published clinical guidelines as can be seen in Australia [22], Canada [23] and UK [23]. Even though it is difficult to create a system that satisfies all stakeholders, the Mongolian pharmaceutical sector needs to expand its assessment processes and report more relevant data in order to allow the analysis of the pharmaceutical sector and develop coordinated systems for decision making in drug reimbursement.

## Limitations

The lack of evidence-based data on private health fund and OOP payment for pharmaceuticals as well as the price components for locally produced or imported medicines, including lack of cost-effectiveness data limit study results to generalize for the pharmaceutical sector in Mongolia.

## Conclusions

Drug pricing and reimbursement of essential medicines is an important task shared by many different authorities, including Ministry of Health and Sport and Ministry of Finance in Mongolia. Periodical reports of the drug registration process, average registration duration and decisions for drug registration are available. However, there is a lack of publicly available reports for the reimbursement decision making process, whereas only information related to drugs that can be reimbursed is made publicly available once consensus has been reached. Price negotiation, budget impact and cost-containment are essential elements for a drug reimbursement process. To allocate resource efficiently in Mongolia is an important and challenging task. Currently the overall system is too complex and there is a lack of accessible data to permit a detailed analysis of the pharmaceutical sector, which is necessary to drive decisions.

## Abbreviations

CIF: Cost, insurance and freight
DOH: Department of Health
DOPS: Department of Physical Culture and Sport
DRF: Drug Revolving Fund
GASI: General Agency for Specialised Inspection
GDP: Gross Domestic Product
GMP: Good manufacturing practice
HI: Health Insurance
HIF: Health Insurance Fund
MNT: Mongolian National Tugrug
MOES: Ministry of Education and Science
MOF: Ministry of Finance, Mongolia
MOH: Ministry of Health of Mongolia
MOHDSW: Ministry of Human Development and Social Welfare
NMPM: National Drug Policy of Mongolia
OOP: Out of pocket
OTC: Over-the-counter
SHIO: Social Health Insurance Office and the
SIGO: Social Insurance General Office
SINC: Social Insurance National Council
THE: Total health expenditure
TPE: Total pharmaceutical expenditure
USD: United States dollar
VAT: Value-Added-Tax
WHO: World Health Organization
